# Gut Microbiota and Nutritional Aspects in Pancreatic Diseases: A Narrative Review

**DOI:** 10.3390/nu18193297

**Published:** 2026-10-08

**Authors:** Klara Szwarc, Joanna Witkowska, Agnieszka Dobrowolska, Agnieszka Zawada

**Affiliations:** 1 Student Scientific Society at the Department of Gastroenterology, Dietetics and Internal Medicine, Poznan University of Medical Sciences, 49 Przybyszewskiego Street, 60-355 Poznan, Poland; 2 Department of General and Transplant Surgery, Poznan University of Medical Sciences, 49 Przybyszewskiego Street, 60-355 Poznan, Poland; 3 Department of Gastroenterology, Dietetics and Internal Medicine, Poznan University of Medical Sciences, 49 Przybyszewskiego Street, 60-355 Poznan, Poland

**Keywords:** gut microbiota, intestinal dysbiosis, pancreatic diseases, intestinal barrier, nutrition

## Abstract

Pancreatic diseases, including acute pancreatitis (AP) and chronic pancreatitis (CP), pancreatogenic diabetes mellitus (T3cDM), autoimmune pancreatitis (AIP), and pancreatic cancer (PDAC), are associated with metabolic, inflammatory, and nutritional disturbances. Increasing evidence indicates a bidirectional relationship between pancreatic diseases and the gut microbiota. On the one hand, alterations in microbial composition and function, intestinal barrier dysfunction, and bacterial translocation may contribute to their pathogenesis and progression. On the other hand, pancreatic diseases and associated changes in nutrition and treatment may affect the composition and function of the gut microbiota. This narrative review aimed to discuss the role of the gut microbiota and nutritional aspects in selected pancreatic diseases. PubMed/MEDLINE was searched up to May 2026 for English-language publications, with emphasis on studies from the previous 10 years, while earlier relevant studies were also considered. Google Scholar was used as a supplementary source. Human observational, experimental and mechanistic studies, as well as secondary literature, were considered, addressing pancreatic microbiota, intestinal permeability, bacterial translocation, nutrition, and microbiota-targeted interventions. Available evidence indicates that pancreatic diseases are associated with alterations in gut and pancreatic microbial communities, although the reported patterns vary between diseases and studies. These changes may be relevant to intestinal barrier integrity, inflammatory signaling, microbial metabolite production, and, in pancreatic cancer, the tumor microenvironment and treatment response. Nutritional management remains an important component of care, particularly in patients with exocrine pancreatic insufficiency and those at risk of malnutrition. However, the causal relationships between pancreatic diseases and microbiota alterations remain incompletely established, and evidence for microbiota-targeted interventions is limited and heterogeneous. Further clinical studies are needed to clarify these interactions and their potential diagnostic and therapeutic relevance.

## 1. Introduction

The pancreas is a principal organ of the gastrointestinal system, performing both exocrine and endocrine functions. Its exocrine role includes the production, synthesis, and secretion of digestive enzymes responsible for breaking down nutrients from food [1]. Acute pancreatitis (AP) can have substantial systemic consequences while persistent or severe pancreatic damage may impair exocrine pancreatic function and nutrient absorption. Destruction of pancreatic cells responsible for endocrine function further contributes to metabolic complications, one of the more serious being the development of pancreatogenic diabetes mellitus (T3cDM). Conversely, newly diagnosed diabetes may also represent the first clinical manifestation of pancreatic carcinoma, particularly in individuals who do not exhibit pronounced features of insulin resistance [2,3].

The intestinal microbiome is a complex community of microorganisms capable of influencing host physiology, including the development of many disorders, particularly gastroenterological and systemic diseases [4,5,6].

Approximately 80% of the gut microbiota consists of bacteria belonging to the *Firmicutes* and *Bacteroidetes* phyla. Significant proportions are also represented by *Proteobacteria*, *Actinobacteria*, *Fusobacteria* and *Verrucomicrobia*, as well as by species of the genus *Actinomyces*. In recent years, scientific interest in gut microbiota alterations has increased substantially, as such changes have been linked to a wide range of diseases. However, data regarding microbiome alterations in pancreatic diseases remain limited [7].

## 2. Aim

The aim of this narrative review was to comprehensively examine the role of the gut microbiota and nutritional aspects in selected pancreatic diseases, including AP, CP, AIP, T3cDM and PDAC. Particular attention was given to the relationship between alterations in gut microbial composition and disease pathogenesis and progression, as well as to the role of nutritional management and selected therapeutic interventions in the course of these diseases.

## 3. Methods

### 3.1. Review Design and Scope

This narrative review focused on the interplay between the gut microbiota, nutritional factors, and selected pancreatic diseases. The scope included alterations in gut and pancreatic microbial communities, intestinal barrier integrity, bacterial translocation, nutritional status and management, and selected microbiota-targeted interventions. Evidence from human observational and interventional studies, experimental models, and relevant secondary literature was considered.

### 3.2. Information Sources and Search Strategy

PubMed/MEDLINE was searched up to May 2026, including English-language publications, primarily those published within the previous 10 years. Earlier publications were considered when they provided important evidence relevant to the topics discussed. Google Scholar was used as a supplementary source to identify additional relevant publications.

The search strategy combined terms related to pancreatic diseases, gut microbiota, and nutritional or microbiota-targeted interventions. Terms related to pancreatic diseases included, among others, “acute pancreatitis”, “chronic pancreatitis”, “type 3c diabetes mellitus”, “pancreatic cancer”, and “pancreatic ductal adenocarcinoma”. Terms related to the microbiota included “gut microbiota”, “gut microbiome”, “intestinal microbiota”, “dysbiosis”, “intestinal dysbiosis”, “intestinal permeability”, “gut barrier”, and “bacterial translocation”. Additional searches included terms related to nutrition and microbiota modulation, such as “diet”, “nutrition”, “dietary intervention”, “probiotics”, “prebiotics”, “synbiotics”, “postbiotics”, and “fecal microbiota transplantation”. Publications relevant to the scope of the review were considered, while studies unrelated to the topics discussed were excluded.

## 4. Pancreatic Microbiota

The pancreas has traditionally been regarded as a sterile organ, and reports concerning the presence of microorganisms within pancreatic tissue remain relatively limited. In recent years, however, bacteria and bacterial genetic material have been detected in pancreatic tissue and pancreatic fluids, raising interest in possible interactions between microorganisms and the pancreas. It remains uncertain whether these findings reflect a stable microbial community residing within the pancreas or, at least in part, transient bacterial translocation and factors related to sample collection and analysis [4,5,7].

Microorganisms may potentially reach the pancreas through several routes, including the duodenum, pancreatic duct, biliary tract, and portal or lymphatic circulation. In particular, disruption of the intestinal barrier may facilitate the translocation of gut-derived bacteria to pancreatic tissue. This process has been proposed as a potential contributor to pancreatic inflammation and the development of complications in certain disease conditions [4,5,7].

The interpretation of microbial findings in pancreatic samples may also be influenced by clinical and procedural factors. Previous pancreatic surgery, biliary drainage, proton pump inhibitor (PPI) use, and the method of sample collection and processing may affect the microbial profiles obtained from pancreatic tissue. Potential contamination or introduction of microorganisms during invasive diagnostic or sampling procedures should also be considered when interpreting these findings [4,8].

In a study by Zwart et al. [8] conducted in patients undergoing pancreatic surgery, bacteria detected in pancreatic biopsies belonged predominantly to *Proteobacteria*, *Firmicutes*, *Actinobacteria*, *Fusobacteria*, *Verrucomicrobia*, and *Bacteroidetes*. Among the most frequently identified taxa were *Enterococcus faecalis*, *Escherichia coli*, and *Enterobacter*-*Klebsiella*. Importantly, the total bacterial load was higher in the pancreatic head than in the tail. The abundance of *Proteobacteria* was also higher in patients who had undergone biliary drainage, while PPI use was associated with a higher overall bacterial load. These findings indicate that microbial profiles detected in pancreatic tissue may be substantially influenced by the anatomical site of sampling and by clinical and treatment-related factors.

### 4.1. Microbial Changes in Acute Pancreatitis

Intestinal barrier dysfunction occurs in approximately 60% of patients with AP and is associated with worse clinical outcomes [7]. Pancreatic cells are able to produce antimicrobial peptides (AMPs), which contribute to the control of microbial growth. During pancreatic injury, AMP levels may decrease, potentially promoting activation of dendritic cells and T lymphocytes, as well as stimulation of macrophages and neutrophils. These processes may contribute to intestinal microbial overgrowth, disruption of intestinal homeostasis, and increased inflammation, potentially influencing the severity of AP [9].

This observation is supported by experimental studies in murine models. Deficiency of cathelicidin-related antimicrobial peptide (CRAMP) in mice lacking calcium release-activated calcium channel protein 1 (Orai1) resulted in bacterial overgrowth, predominantly driven by an expansion of Proteobacteria. This dysbiosis was associated with intestinal inflammation characterized by prominent lymphocytic infiltration and ultimately death within three weeks. Supplementation with CRAMP-containing agents prevented these adverse outcomes [9]. Experimental data from Ahuja et al. [9] suggest that pancreatic antimicrobial secretion contributes to the maintenance of intestinal microbial homeostasis. Sun et al. [10] demonstrated in a murine model of autoimmune diabetes that short-chain fatty acids produced by the gut microbiota promoted CRAMP production by pancreatic β-cells. This pathway enhanced regulatory immune responses within pancreatic islets and reduced local inflammation, suggesting a link between gut microbial metabolites and the pancreatic immune environment.

Zhao et al. [11] demonstrated distinct alterations in the pancreatic and ileal microbiota during experimental AP, including an expansion of *Escherichia*–*Shigella* in pancreatic tissue and changes in several other bacterial taxa, suggesting possible microbial translocation between the gut and pancreas. These findings indicate that pancreatic microbial profiles may change during AP, although experimental observations should not be interpreted as definitive evidence of a stable resident pancreatic microbiota.

Furthermore, Zwart et al. [8] reported a weak correlation between the microbial profiles of pancreatic tissue and those detected in duodenal samples, although the duodenal microbiome did not adequately reflect the pancreatic microbiome. Li et al. [12] detected several bacterial genera in pancreatic cyst fluid, including *Acidaminococcus*, *Bacteroides*, *and Escherichia*/*Shigella*. Consistent findings have been reported in murine models. Following administration of fluorescently labeled *Enterococcus faecalis*, bacteria were subsequently detected in pancreatic tissue, suggesting gut-to-pancreas bacterial translocation. *Escherichia coli* was also detected in the pancreatic tissue of mice with severe acute pancreatitis (SAP) but was absent in healthy controls. These observations are consistent with experimental findings showing translocation of *Escherichia coli* from the gut to pancreatic tissue following induction of AP [13].

Collectively, these findings suggest that translocation of gut-derived bacteria to pancreatic tissue may be involved in the pancreatic response to intestinal barrier disruption, although its contribution to the initiation and progression of AP remains uncertain [4,5,11].

### 4.2. Microbial Alterations in Chronic Pancreatitis

In patients with pancreatic calcification associated with chronic pancreatitis (CP), increased levels of bacteria belonging to the *Proteobacteria phylum* and endotoxins have been detected within the pancreatic duct, whereas healthy controls exhibited higher levels of *Lactobacillus* [8]. The presence of endotoxins was also associated with an increased risk of diabetes among individuals with CP. Brubaker et al. [14] reported a significant negative correlation between the relative abundance of *Faecalibacterium prausnitzii* and plasma endotoxin concentrations, in contrast to a positive correlation between the relative abundance of *Shigella* and plasma endotoxin levels. These findings suggest that specific components of the gut microbiota may be associated with inflammatory and endotoxin-related features in patients with CP [14].

This concept is further supported by the study of Demir et al. [15], which examined patients with CP undergoing pancreatic surgery and demonstrated a substantial bacterial load in pancreatic fistula fluid, with *Klebsiella pneumoniae* (27%) and *Escherichia coli* (55%) being the most frequently identified bacteria. These findings indicate that microorganisms may be detected in pancreatic-associated fluids in patients with CP; however, they do not by themselves establish the presence of a stable resident pancreatic microbial community.

### 4.3. Microbial Alterations in Autoimmune Pancreatitis

Experimental evidence suggests that NOD1 (nucleotide-binding oligomerization domain-containing protein 1), an intracellular pattern-recognition receptor that detects bacterial ligands, may be involved in the development of non-infectious pancreatitis. Activation of NOD1 occurs within pancreatic acinar cells and may be triggered by physiological microorganisms or microbial components translocated from the intestine [16].

A key function of NOD1 is the upregulation of pro-inflammatory mediators, including interleukin 6 (IL-6) and tumor necrosis factor α (TNF-α). This response is followed by the recruitment of neutrophils and macrophages into the pancreatic parenchyma and may contribute to trypsin activation and initiation of the zymogen cascade, ultimately leading to further leukocyte activation. Collectively, these processes may result in systemic inflammatory response syndrome (SIRS) and multiorgan failure [5,16]. Moreover, correlations have been reported between circulating IL-6 levels, the presence of bacteria belonging to the *Enterobacteriaceae* family and *Enterococcus* genus, and reduced abundance of bacteria belonging to the genus *Bifidobacterium* [17].

In the pathogenesis of autoimmune pancreatitis (AIP), increasing attention has been directed toward the potential role of gut dysbiosis. A central immunological feature of AIP involves an enhanced IgG4-mediated immune response, driven in part by activation of plasmacytoid dendritic cells (pDCs). Activated pDCs produce interferon α (IFN-α) and interleukin 33 (IL-33), ultimately contributing to a fibro-inflammatory response within the pancreas. Gut dysbiosis may also contribute to pDC activation, eliciting downstream cytokine-mediated pathways and potentially reinforcing the inflammatory and fibrotic processes characteristic of AIP [18,19].

Among the microbial disturbances associated with AIP, particular attention has been given to *Klebsiella pneumoniae* strains, which may activate plasmacytoid dendritic cells. Additionally, short-chain fatty acids (SCFAs) and bile acids may contribute to disease pathogenesis by inducing activation of M2 macrophages, which subsequently produce IL-33. The resulting expansion of M2 macrophages, together with activated pDCs, may be associated with enhanced immune activation, fibrosis, and loss of pancreatic acinar cells [19]. These findings primarily concern interactions between the gut microbiota and the pancreatic immune environment rather than providing evidence for a stable resident microbiota within the pancreas.

**Figure 1:**
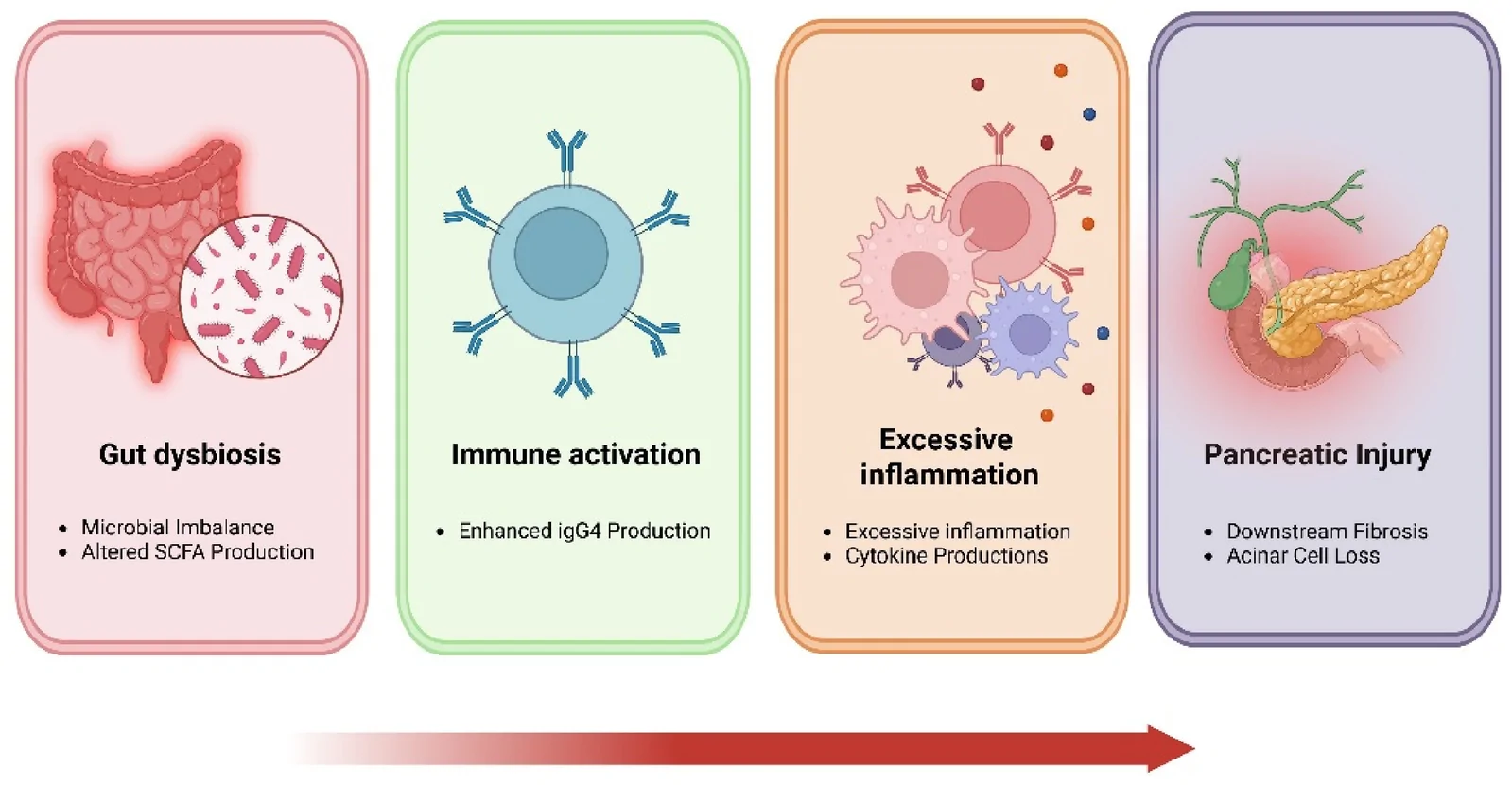
Potential pathway linking gut dysbiosis to pancreatic injury in autoimmune pancreatitis (AIP).

A potential pathway linking gut dysbiosis to pancreatic injury in AIP is illustrated in Figure 1.

### 4.4. Microbial Findings in Pancreatic Cancer

Geller et al. [20] detected bacteria in 76% of human pancreatic ductal adenocarcinoma (PDAC) samples, with Gammaproteobacteria among the predominant bacterial groups identified. Their findings further indicate that intratumoral bacteria can metabolize gemcitabine and may thereby contribute to reduced therapeutic efficacy.

Pushalkar et al. [21] further demonstrated that the pancreatic tumor microbiome can promote tumorigenesis and influence tumor progression in experimental models.

*Escherichia coli* and members of the genus Pseudomonas have also been detected in pancreatic head tumors [4,22].

Patients with PDAC have also been reported to exhibit increased abundance of bacteria belonging to the *Proteobacteria* phylum, accounting for nearly half of the detected bacterial taxa, as well as members of the Gammaproteobacteria class, including *Acinetobacter*, *Enterobacter*, *Delftia*, *Klebsiella* and *Sphingomonas*. Increased abundances of members of the *Firmicutes* and *Fusobacteria* phyla and genera belonging to *Actinobacteria*, including *Corynebacterium* and *Propionibacterium* have also been reported. In contrast, *Lactobacillus* levels were reduced compared with healthy controls. Additionally, higher intratumoral levels of *Malassezia* species have been associated with PDAC progression, whereas *Candida*, *Aspergillus*, and *Saccharomyces* did not promote tumor development following recolonization after microbiome ablation [23].

The diversity and composition of microbial profiles detected within pancreatic tumors may also be associated with clinical outcomes. In a study comparing patients with PDAC who had shorter versus longer survival, differences in the intratumoral microbial profiles were associated with long-term survival [23]. However, these findings should be interpreted in the context of the study population and methodology. In particular, Chakladar et al. [24] analyzed inferred microbial abundance from RNA-sequencing data and reported associations with carcinogenesis, metastasis, immune suppression, sex, and smoking status rather than demonstrating a causal relationship between a resident pancreatic microbiota and these outcomes.

Higher intratumoral microbial abundance has also been associated with immunosuppression and adverse clinical outcomes, including increased metastatic potential and reduced overall survival [22,23]. The presence of *Acidovorax ebreus*, *Citrobacter freundii*, *Shigella sonnei*, members of the order *Pseudomonadales*, and members of the class *Betaproteobacteria* has been correlated with adverse clinical outcomes and increased risk of cancer progression [24].

These microbial alterations may have functional consequences for the tumor microenvironment and immune regulation, although their clinical significance remains to be established [21,23,24].

## 5. The Relationship Between Pancreatic Disorders and Intestinal Dysbiosis

The available evidence is presented according to study type, distinguishing findings from human studies, experimental and mechanistic evidence, and secondary literature. Experimental findings are interpreted as complementary mechanistic evidence rather than as direct evidence of clinical effects in patients with pancreatic diseases.

### 5.1. Acute Pancreatitis

Secondary literature. AP may promote ischemic injury within the intestinal mucosa, ultimately leading to disruption of gut barrier integrity, which in turn exacerbates the ongoing inflammatory response in the pancreas [5]. Zhang et al. [25] reported that patients with AP had higher abundances of *Proteobacteria* and *Bacteroidetes* and lower abundances of *Firmicutes* and *Actinobacteria* compared with healthy controls. Zhu et al. [26] further observed increased abundances of *Escherichia*/*Shigella*, *Enterococcus*, and *Enterobacteriaceae* in patients with AP, while *Bacteroides*, *Blautia*, and other commensal taxa were reduced in specific AP severity groups. Tan et al. [17] reported an increase in *Enterococcus* and a decrease in *Bifidobacterium* with increasing disease severity. Overall, these studies suggest that gut microbiota composition differs between patients with AP and healthy controls, although the direction and specific taxonomic changes are not fully consistent across studies.

However, in contrast to these findings, Patel et al. [5] reported a decreased abundance of bacteria belonging to the *Bacteroidetes* phylum, accompanied by an increased abundance of *Firmicutes*. These discrepancies may reflect differences in disease severity, geographical and dietary backgrounds, timing of stool sample collection, previous antibiotic exposure, sequencing methods, taxonomic resolution, and bioinformatic pipelines. Therefore, differences in study populations and methodological approaches should be considered when interpreting the variability of microbiota findings in AP [7,14].

Human observational evidence. Similarly, in patients with SAP and dysbiosis, elevated levels of interleukin 6 (IL-6) have been observed, accompanied by increased abundance of bacteria from the *Enterobacteriaceae* family and the *Enterococcus* genus, along with a reduction in *Bifidobacterium* spp. Notably, the presence of *Enterococcus* has been shown to correlate with circulating endotoxin levels in plasma [17].

### 5.2. Autoimmune and Chronic Pancreatitis

Secondary literature. Yoshikawa et al. [19] reported that gut microbiota profiles differed between patients with AIP and CP, with higher proportions of *Bacteroides*, *Streptococcus*, and *Clostridium* species observed in patients with CP.

Human observational evidence. Frost et al. [27] reported that patients with CP exhibited markedly reduced intestinal microbial diversity, accompanied by increased abundances of *Streptococcus*, *Enterococcus*, and *Escherichia*–*Shigella*. They also observed a reduced abundance of bacteria associated with SCFA production, including *Faecalibacterium*. Notably, these microbiota alterations were independent of exocrine pancreatic insufficiency.

These changes may have functional implications for intestinal homeostasis. The increased abundance of *Enterococcus* and *Escherichia*–*Shigella* may reflect a shift toward opportunistic or potentially pro-inflammatory microbial profiles, whereas the reduction in *Faecalibacterium* may indicate a loss of bacteria involved in SCFA production and intestinal barrier maintenance. Similarly, alterations in *Bacteroides* and *Clostridium* may affect microbial fermentation and metabolite production, although the clinical and causal significance of these taxonomic changes in CP remains uncertain [19,27].

Adolph et al. [28] also summarized experimental evidence suggesting that microbiota-derived metabolites may influence AIP. Experimental studies discussed in this review have reported that sodium butyrate can delay or reduce the development of type 1 diabetes in experimental models. These findings provide mechanistic evidence supporting a potential role of SCFA-mediated pathways; however, they should not be considered equivalent to direct clinical evidence in patients with type 1 diabetes.

### 5.3. Chronic Alcoholic Pancreatitis

Human observational evidence. Ciocan et al. [29] reported that patients with chronic alcoholic pancreatitis (CAP) had lower intestinal microbial diversity and a different overall microbiota composition compared with alcoholic controls. The microbiota of patients with CAP showed increased abundances of *Klebsiella*, *Enterococcus*, and *Sphingomonas*. Jandhyala et al. [30] reported a reduction in the abundances of *Faecalibacterium prausnitzii* and *Ruminococcus bromii* in patients with CP, accompanied by an increased *Firmicutes*-to-*Bacteroidetes* ratio.

These microbial alterations may be associated with impaired intestinal barrier function and increased exposure to microbial products in patients with CP, although causal relationships remain uncertain. The reduction in *Faecalibacterium prausnitzii*, a bacterium associated with intestinal barrier integrity, has been linked to increased circulating endotoxin levels, while reduced *Ruminococcus bromii* may affect starch degradation, microbial metabolism, and carbohydrate fermentation. Overall, these findings suggest that dysbiosis in CP may be associated with impaired intestinal barrier function, increased exposure to microbial products, and metabolic alterations, although causal relationships remain uncertain [30].

Alcohol consumption itself may influence gut microbiota composition and therefore represent an important potential confounding factor when interpreting microbiota alterations in patients with alcoholic pancreatitis [29].

### 5.4. Pancreatic Cancer

Human and tissue-based evidence. Pushalkar et al. [21] reported that the intrapancreatic microbiome of patients with pancreatic ductal adenocarcinoma (PDAC) was dominated by *Proteobacteria*, *Bacteroidetes*, and *Firmicutes*, which accounted for approximately 45%, 31%, and 22% of the bacterial community, respectively. *Proteobacteria*, particularly Gammaproteobacteria, were also enriched in the gut microbiota of patients with PDAC compared with healthy controls [31]. Li et al. [12] reported that bacterial DNA was detected in 98.2% of pancreatic cyst fluid samples, with *Bacteroides*, *Escherichia*/*Shigella*, and *Acidaminococcus* among the predominant genera; *Staphylococcus* and *Fusobacterium* were also detected.

However, these findings demonstrate associations between PDAC and microbial alterations but do not establish whether such changes precede disease development, result from the disease or its treatment, or reflect disease severity [21].

The main changes in gut microbiota reported in individuals with pancreatic diseases are summarized in Table 1. The table highlights disease-specific alterations in microbial composition and diversity reported across the available studies.

Importantly, observed differences in gut microbiota composition may also be influenced by potential confounding factors, including antibiotic and other medication use, dietary patterns, hospitalization, disease severity, exocrine pancreatic insufficiency, alcohol consumption, and smoking. Therefore, microbiota alterations should be interpreted cautiously and should not be considered specific causal signatures of pancreatic disease without accounting for these factors [27,29,35].

## 6. Therapeutic Approaches Targeting the Gut Microbiota in Pancreatic Diseases

The use of antibiotics, which are commonly administered in severe forms of AP, has an adverse impact on gut microbiota composition, as it may lead to depletion of the gastrointestinal microbial community. In contrast, early initiation of enteral nutrition exerts a protective effect on the intestinal microbiota [5].

The use of probiotics and synbiotics has been investigated in both AP and CP. Patel et al. [5] described potential beneficial effects of probiotics containing *Faecalibacterium* and *Bifidobacterium* species, including stabilization of the intestinal barrier, enhancement of anti-inflammatory responses, and attenuation of bacterial translocation. However, clinical evidence in AP remains inconsistent. Besselink et al. [36] conducted a randomized, double-blind, placebo-controlled trial in patients with predicted severe AP and found that a multispecies probiotic preparation did not reduce infectious complications and was associated with an increased risk of mortality. In contrast, the meta-analysis by Gou et al. [37] found no significant difference in mortality across the included trials. These discrepancies may be related to differences in probiotic strains and combinations, treatment protocols, patient populations, disease severity, and treatment duration.

Fecal microbiota transplantation (FMT) represents another approach aimed at modifying the intestinal microbiota and restoring microbial homeostasis. However, evidence regarding its use in pancreatic diseases remains limited and differs according to the type of evidence available.

Clinical evidence is limited to individual reports. Hu et al. [38] described the use of FMT in a patient with pancreatitis complicated by severe *Clostridioides difficile* infection, with clinical improvement following transplantation. However, FMT was primarily used to treat severe *C. difficile* infection rather than pancreatitis itself.

Preclinical evidence provides additional insights into the potential effects of FMT in PDAC Riquelme et al. [23] investigated FMT in a murine model using fecal material obtained from patients with PDAC with different survival outcomes. FMT modified the tumor-associated microbiota in recipient mice and was associated with differences in tumor growth and immune-cell infiltration. Mice receiving microbiota from long-term survivors showed reduced tumor growth, increased CD8+ T-cell activity, and improved survival compared with mice receiving microbiota from short-term survivors. These findings suggest that the gut microbiota may influence the pancreatic tumor microenvironment, although their clinical relevance remains to be established.

Furthermore, Pushalkar et al. [21] and Li et al. [39] summarized experimental evidence suggesting that modulation of the gut microbiota may influence pancreatic tumor development through changes in inflammatory and immune responses. However, direct clinical evidence that FMT can reduce tumor growth, improve treatment response, or prolong survival in patients with PDAC remains insufficient. FMT should therefore currently be regarded as an investigational approach rather than an established therapeutic intervention in PDAC.

Nonetheless, due to the potential for more controlled modulation of the microbiota, a new generation of microbiota-based therapies has been proposed, involving selected microbial components, such as specific bacterial strains or defined microbial consortia, rather than whole fecal material. Such approaches may provide greater control over the composition of the administered microbiota; however, their safety, reproducibility, and therapeutic efficacy in pancreatic diseases remain to be established.

Li et al. [39] summarized evidence indicating that the gut microbiota may influence treatment outcomes in cancer. In PDAC, microbial alterations have been associated with drug sensitivity, the tumor immune microenvironment, and tumor metabolism; however, the clinical utility of microbiome profiling for diagnostic or therapeutic purposes remains investigational.

Microbial metabolism may also influence the efficacy of anticancer therapy. Geller et al. [20] reported that intratumoral Gammaproteobacteria can metabolize gemcitabine into an inactive metabolite, thereby contributing to gemcitabine resistance. These findings suggest that modulation of bacteria contributing to drug resistance may represent a potential strategy for improving treatment response; however, the clinical applicability of such approaches remains to be established.

## 7. The Influence of Dietary and Lifestyle Modifications on the Gut Microbiota in Pancreatic Disorders

Lifestyle factors, particularly alcohol consumption and diet, can influence gut microbiota composition and may be relevant to the development and progression of pancreatic diseases. A study conducted in mice demonstrated that alcohol intake increased the relative abundance of *Verrucomicrobia*, *Actinobacteria*, and *Proteobacteria*. In contrast, excessive alcohol consumption resulted in a reduction in bacteria belonging to the *Firmicutes* phylum and the genus *Lactobacillus*, an increase in *Enterococcus*, and an overall higher risk of dysbiosis [40]. Acetaldehyde, a toxic metabolite generated during ethanol metabolism, may further impair pancreatic function by promoting the excessive secretion of inflammatory mediators, thereby increasing the risk of CP. These alcohol-associated microbial alterations may contribute to intestinal barrier dysfunction and increased exposure to microbial products, potentially providing an additional pathway linking alcohol consumption with pancreatic inflammation; however, the available evidence is predominantly experimental [41].

Furthermore, an unhealthy diet and lifestyle may affect pancreatic secretion and digestive processes. Impaired digestion may subsequently contribute to disturbances in the intestinal microbiota, and may facilitate bacterial translocation, potentially affecting the pancreatic and ductal microbiota. Such alterations in the pancreatic microenvironment may exacerbate inflammation and potentially contribute to the development or progression of pancreatic diseases, including PDAC [4,5].

In cases of long-term adipose tissue accumulation, a reduced diversity of the gut microbiota has been observed, including a decline in *Bacteroidetes* and an increase in *Firmicutes*, changes that may be driven by insufficient dietary fiber intake [42].

Mediterranean dietary patterns are characterized by a high intake of plant-based foods and dietary fiber and have been associated with favorable changes in gut microbial composition [43]. The Mediterranean diet has been associated with changes in the abundance of several bacterial taxa, including *Roseburia*, *Blautia*, *Prevotella*, and *Faecalibacterium prausnitzii* [44].

*Akkermansia muciniphila* has been associated with improved intestinal barrier integrity and modulation of inflammatory responses [45]. Furthermore, the Mediterranean diet is rich in microbiota-accessible carbohydrates, which lead to increased production of SCFAs in the colon and stool. SCFAs are major products of microbial fermentation of dietary fiber and play an important role in maintaining intestinal barrier function and immune homeostasis [46].

*Bacteroides* species, which primarily produce propionate and acetate, together with *Firmicutes*, which generate butyrate, are the main contributors to SCFA production. Fermentation of dietary fiber into SCFAs may support microbial diversity and intestinal homeostasis. In addition, SCFAs play a key role in maintaining the homeostasis and function of intestinal epithelial cells [46].

Prebiotics are selectively fermented substrates that can modify the composition and activity of the gut microbiota and promote the production of microbial metabolites such as SCFAs [47,48]. Resistant starch is one example of a prebiotic substrate that can enhance SCFA production. Because prebiotic substrates can promote SCFA production and support intestinal barrier function, they may be relevant to pancreatic diseases in which intestinal dysbiosis and barrier dysfunction have been implicated; however, direct clinical evidence remains limited [4,46,47].

## 8. Nutrition in Pancreatic Diseases

### 8.1. Pancreatogenic Diabetes Mellitus

Dietary management of T3cDM should be individualized and accompanied by nutritional education. T3cDM may coexist with pancreatic exocrine insufficiency (PEI), requiring pancreatic enzyme replacement therapy (PERT). Enzyme replacement therapy improves the digestion and absorption of nutrients, reduces the risk of malnutrition, and may also contribute to improved glycemic stability [49,50]. Excessive energy restriction is not recommended in patients with T3cDM because of the risk of malnutrition, loss of muscle mass (sarcopenia), and deterioration of glycemic control. Therefore, energy, protein, fat, and carbohydrate intake should be individually tailored by a qualified dietitian [51,52].

Importantly, routine severe restriction of dietary fat is not recommended, as it may increase the risk of malnutrition and fat-soluble vitamin deficiencies and may exacerbate steatorrhea. Protein-rich foods and, when appropriate, medium-chain triglycerides (MCTs), may be considered in patients with impaired fat digestion [49,50].

The American Diabetes Association (ADA) does not recommend a single specific dietary pattern or macronutrient distribution for patients with pancreatic diabetes. Instead, nutritional management should be individualized according to metabolic goals, nutritional status, comorbidities, treatment, and patient preferences. Dietary patterns emphasizing vegetables and fruits, whole-grain products, lean protein sources, and minimally processed foods may be considered, while the intake of added sugars, sugar-sweetened beverages, and highly processed foods should be limited [49,53].

### 8.2. Autoimmune Pancreatitis

Vujasinovic et al. [54] reported that 38% of patients with AIP developed at least one micronutrient deficiency during a median follow-up of 53 months. Zinc deficiency (25.5%) and vitamin D deficiency (16.1%) were the most frequently observed. Importantly, patients with PEI remained at increased risk of micronutrient deficiencies despite pancreatic enzyme replacement therapy. These findings highlight the importance of monitoring nutritional status and selected micronutrient levels in patients with AIP, particularly in those with PEI.

### 8.3. Acute Pancreatitis

In patients with mild AP, oral feeding, if well tolerated, should be initiated as early as possible, regardless of serum lipase levels. When oral feeding is not tolerated, enteral nutrition (EN) should be initiated within 24–72 h of hospital admission, with nasogastric feeding generally preferred and nasojejunal feeding considered in cases of intolerance [52]. Compared with total parenteral nutrition (TPN), EN is associated with lower mortality and a reduced risk of infectious complications and multiple organ failure [55]. These beneficial effects may be partly related to the preservation of intestinal barrier integrity and reduction in bacterial translocation, thereby potentially limiting disturbances in the gut microbiota. However, evidence regarding the optimal timing of EN remains heterogeneous, particularly in patients with mild or moderately severe disease [52,55].

Early initiation of EN within 24 h of admission has also been investigated in patients with predicted severe AP [56]. Bakker et al. [57] found no significant reduction in major infection or mortality when EN initiated within 24 h was compared with an oral diet initiated after 72 h. Therefore, the timing of nutritional support should be considered in relation to disease severity and tolerance to oral feeding [56,57].

Parenteral nutrition should be reserved for patients in whom EN is contraindicated or fails to meet nutritional requirements. Routine pancreatic enzyme supplementation is not indicated unless pancreatic exocrine insufficiency (PEI) is confirmed [52].

Individual randomized controlled trials have suggested potential benefits of omega-3 fatty acid supplementation on selected inflammatory and clinical parameters in patients with AP; however, the findings remain inconclusive, and current evidence is insufficient to support routine supplementation [58]. Similarly, current evidence does not support the routine use of probiotics or glutamine in AP [52].

### 8.4. Chronic Pancreatitis

Among patients with CP, Arvanitakis et al. [52] recommend a balanced diet without routine fat restriction, while in malnourished individuals, energy and protein intake should be increased through more frequent, smaller meals. Restrictive diets are not recommended, except when individualized dietary adjustments are required.

The most common causes of malnutrition and reduced food intake include disease-related abdominal pain, gastrointestinal symptoms, and pancreatic exocrine and endocrine insufficiency. Loss of pancreatic exocrine function leads to impaired digestion and nutrient absorption and may be further influenced by chronic inflammation, diabetes, smoking, and alcohol consumption [59].

Patients with CP should be screened for malnutrition risk and monitored for osteoporosis and macro- and micronutrient deficiencies [50]. Deficiencies of fat-soluble vitamins, vitamin B12, folate, thiamine, and minerals such as calcium, zinc, selenium, magnesium, and iron may occur [52]. When the usual diet does not meet energy and protein requirements, oral nutritional supplements (ONS) are recommended. In cases of persistent malabsorption despite adequate pancreatic enzyme replacement therapy, formulations containing medium-chain triglycerides (MCTs) may be considered. For patients who continue to fail to meet their nutritional targets, EN should be initiated [52,60].

### 8.5. Pancreatic Cancer

PDAC is associated with a high risk of malnutrition, weight loss, and cancer cachexia; therefore, nutritional status should be assessed at diagnosis and regularly throughout treatment [61]. Patients undergoing pancreaticoduodenectomy should also be assessed for sarcopenia, which may be associated with treatment outcomes [62]. Nutritional interventions include dietary counseling, oral nutritional supplements (ONS), enteral nutrition (EN), and parenteral nutrition (PN) [61,62].

In patients with PDAC, oral feeding should be preferred when tolerated, with dietary modification initially focusing on increasing energy and protein intake. If oral intake remains inadequate, ONS may be added, followed by EN when oral intake is insufficient or impossible. PN should be reserved for situations in which the gastrointestinal tract cannot be used or its function is significantly impaired [61,63]. In patients with PDAC and PEI, pancreatic enzyme replacement therapy is also recommended [50].

Emanuel et al. [61] noted that omega-3 fatty acid supplementation may help maintain or increase lean body mass in selected patients with cancer; however, evidence regarding its impact on clinical outcomes remains insufficient. Similarly, the efficacy of other immunomodulatory strategies, such as glutamine or arginine supplementation, requires further investigation.

Special dietary patterns, including ketogenic, Mediterranean, and low-sugar diets, are increasingly being investigated in PDAC; however, available evidence is still limited, with much of it derived from preclinical studies, and these diets cannot currently be routinely recommended [63].

## 9. Conclusions

Available evidence indicates that pancreatic disorders are associated with alterations in gut and pancreatic microbial composition, although the specific taxonomic patterns vary across diseases and studies.

Patients with AP have been reported to show increased abundances of *Enterococcus*, *Escherichia*–*Shigella*, and members of the *Enterobacteriaceae* family, together with reduced abundances of *Bifidobacterium* and bacteria belonging to the *Firmicutes* and *Actinobacteria* phyla.

Patients with CP have been reported to exhibit increased abundances of *Streptococcus*, *Bacteroides*, *Clostridium*, and *Enterococcus*, together with reduced abundances of *Faecalibacterium prausnitzii* and *Ruminococcus bromii*.

In AP and CP, these microbiota alterations may be relevant to intestinal barrier integrity, exposure to endotoxins and other microbial products, inflammatory signaling, and microbial metabolite production. However, the specific biological and clinical significance of individual taxonomic changes, as well as their potential causal contribution to disease development or progression, remain uncertain.

In PDAC, alterations in gut and intratumoral microbial communities may additionally influence the tumor immune microenvironment and treatment response, including through microbial metabolism of anticancer agents. However, these potential mechanisms remain incompletely understood and require further validation in well-characterized clinical studies.

Taken together, these findings suggest that disease-associated microbial patterns may have potential as biomarkers of pancreatic diseases. However, their clinical utility for risk assessment or early diagnosis remains uncertain and requires prospective validation in well-characterized patient populations. Future research should focus on identifying reproducible microbial signatures and determining their diagnostic value, as well as exploring the relationship between the gut and pancreatic microbiomes and potential therapeutic targets.

## Figures and Tables

**Table 1:** Changes in gut microbiota in individuals with pancreatic diseases.

Author, Year	Disease	Material	Increased Bacterial Population	Decreased Bacterial Population
S. Yu, 2020 [32]	AP	Fecal samples	*Bacteroides*, *Escherichia*–*Shigella*, *Enterococcus*	-
Y. Zhu, 2019 [26]	AP	Fecal samples	-	*Blautia and Bifidobacterium*
Zhang, 2018 [25]	AP	Fecal samples	*Bacteroidetes*, *Proteobacteria*,	*Firmicutes*, *Actinobacteria*
Tan, 2015 [17]	AP	Fecal samples	*Enterobacteriaceae*, *Enterococcus*	*Bifidobacterium*
Patel, 2021 [5]	AP	Fecal samples	*Firmicutes*	*Bacteroidetes*
Zhou et al., 2020 [33]	CP	Fecal samples	*Proteobacteria*	*Firmicutes*, *Actinobacteria*
F. Frost, 2020 [27]	CP	Fecal samples	*Enterococcus*, *Streptococcus*, *Escherichia*–*Shigella*	-
Jandhyala et al., 2017 [30]	CP	Fecal samples	*Firmicutes*, *Bacteroidetes*	*Faecalibacterium prausnitzii*, *Ruminococcus bromii*
Aykut et al., 2019 [22]	PDAC	Fecal samples	*Ascomycota*, *Basidiomycota*, *Malassezia*	-
Half, 2019 [34]	PDAC	Fecal samples	*Bacteroides, Verrucomicrobia*, *Sutterella*, *Veillonella*, *Bacteroides*, *Odoribacter*, *Akkermansia*	*Firmicutes*, *Actinobacteria*
Pushalkart, 2018 [21]	PDAC	Fecal samples	*Proteobakteria*, *Actinobacteria*, *Fusobacteria*, *Verrucomicrobia*	-

AP (*Acute Pancreatitis*), CP (*Chronic Pancreatitis*), PDAC (*Pancreatic cancer*).

## Data Availability

No new data were created or analyzed in this study. Data sharing is not applicable to this article.

## References

[1] Habtezion A., Gukovskaya A.S., Pandol S.J. (2019). Acute Pancreatitis: A Multifaceted Set of Organelle and Cellular Interactions. Gastroenterology.

[2] Ewald N., Bretzel R.G. (2013). Diabetes Mellitus Secondary to Pancreatic Diseases (Type 3c)—Are We Neglecting an Important Disease?. Eur. J. Intern. Med..

[3] Gallo M., Adinolfi V., Morviducci L., Acquati S., Tuveri E., Ferrari P., Zatelli M.C., Faggiano A., Argentiero A., Natalicchio A. (2021). Early Prediction of Pancreatic Cancer from New-Onset Diabetes: An Associazione Italiana Oncologia Medica (AIOM)/Associazione Medici Diabetologi (AMD)/Società Italiana Endocrinologia (SIE)/Società Italiana Farmacologia (SIF) Multidisciplinary Consensus Position Paper. ESMO Open.

[4] Schepis T., De Lucia S.S., Nista E.C., Manilla V., Pignataro G., Ojetti V., Piccioni A., Gasbarrini A., Franceschi F., Candelli M. (2021). Microbiota in Pancreatic Diseases: A Review of the Literature. J. Clin. Med..

[5] Patel B.K., Patel K.H., Bhatia M., Iyer S.G., Madhavan K., Moochhala S.M. (2021). Gut Microbiome in Acute Pancreatitis: A Review Based on Current Literature. World J. Gastroenterol..

[6] Cen M.E., Wang F., Su Y., Zhang W.J., Sun B., Wang G. (2018). Gastrointestinal Microecology: A Crucial and Potential Target in Acute Pancreatitis. Apoptosis.

[7] Akshintala V.S., Talukdar R., Singh V.K., Goggins M. (2019). The Gut Microbiome in Pancreatic Disease. Clin. Gastroenterol. Hepatol..

[8] Zwart E.S., Jeleniewski S., Boyd L.N.C., Meijer L.L., Puik J.R., Zonderhuis B.M., Daams F., Budding A.E., Mebius R.E., Kazemier G. (2022). Factors That Influence the Pancreatic and Duodenal Microbiome in Patients Undergoing Pancreatic Surgery. PLoS ONE.

[9] Ahuja M., Schwartz D.M., Tandon M., Son A., Zeng M., Swaim W., Eckhaus M., Hoffman V., Cui Y., Xiao B. (2017). Orai1-Mediated Antimicrobial Secretion from Pancreatic Acini Shapes the Gut Microbiome and Regulates Gut Innate Immunity. Cell Metab..

[10] Sun J., Furio L., Mecheri R., van der Does A.M., Lundeberg E., Saveanu L., Chen Y., van Endert P., Agerberth B., Diana J. (2015). Pancreatic β-Cells Limit Autoimmune Diabetes via an Immunoregulatory Antimicrobial Peptide Expressed under the Influence of the Gut Microbiota. Immunity.

[11] Zhao M., Cui M., Jiang Q., Wang J., Lu Y. (2023). Profile of Pancreatic and Ileal Microbiota in Experimental Acute Pancreatitis. Microorganisms.

[12] Li S., Fuhler G.M., Bn N., Jose T., Bruno M.J., Peppelenbosch M.P., Konstantinov S.R. (2017). Pancreatic Cyst Fluid Harbors a Unique Microbiome. Microbiome.

[13] Samel S., Lanig S., Lux A., Keese M., Gretz N., Nichterlein T., Sturm J., Löhr M., Post S. (2002). The Gut Origin of Bacterial Pancreatic Infection during Acute Experimental Pancreatitis in Rats. Pancreatology.

[14] Brubaker L., Luu S., Hoffman K.L., Wood A., Navarro Cagigas M., Yao Q., Petrosino J.F., Fisher W., Van Buren G. (2021). Microbiome Changes Associated with Acute and Chronic Pancreatitis: A Systematic Review. Pancreatology.

[15] Demir E., Abdelhai K., Demir I.E., Jäger C., Scheufele F., Schorn S., Rothe K., Friess H., Ceyhan G.O. (2020). Association of Bacteria in Pancreatic Fistula Fluid with Complications after Pancreatic Surgery. BJS Open.

[16] Tsuji Y., Watanabe T., Kudo M., Arai H., Strober W., Chiba T. (2012). Sensing of Commensal Organisms by the Intracellular Sensor NOD1 Mediates Experimental Pancreatitis. Immunity.

[17] Tan C., Ling Z., Huang Y., Cao Y., Liu Q., Cai T., Yuan H., Liu C., Li Y., Xu K. (2015). Dysbiosis of Intestinal Microbiota Associated with Inflammation Involved in the Progression of Acute Pancreatitis. Pancreas.

[18] Kamata K., Watanabe T., Minaga K., Hara A., Yoshikawa T., Okamoto A., Yamao K., Takenaka M., Park A.-M., Kudo M. (2019). Intestinal Dysbiosis Mediates Experimental Autoimmune Pancreatitis via Activation of Plasmacytoid Dendritic Cells. Int. Immunol..

[19] Yoshikawa T., Watanabe T., Kamata K., Hara A., Minaga K., Kudo M. (2021). Intestinal Dysbiosis and Autoimmune Pancreatitis. Front. Immunol..

[20] Geller L.T., Barzily-Rokni M., Danino T., Jonas O.H., Shental N., Nejman D., Gavert N., Zwang Y., Cooper Z.A., Shee K. (2017). Potential Role of Intratumor Bacteria in Mediating Tumor Resistance to the Chemotherapeutic Drug Gemcitabine. Science.

[21] Pushalkar S., Hundeyin M., Daley D., Zambirinis C.P., Kurz E., Mishra A., Mohan N., Aykut B., Usyk M., Torres L.E. (2018). The Pancreatic Cancer Microbiome Promotes Oncogenesis by Induction of Innate and Adaptive Immune Suppression. Cancer Discov..

[22] Aykut B., Pushalkar S., Chen R., Li Q., Abengozar R., Kim J.I., Shadaloey S.A., Wu D., Preiss P., Verma N. (2019). The Fungal Mycobiome Promotes Pancreatic Oncogenesis via Activation of MBL. Nature.

[23] Riquelme E., Zhang Y., Zhang L., Montiel M., Zoltan M., Dong W., Quesada P., Sahin I., Chandra V., San Lucas A. (2019). Tumor Microbiome Diversity and Composition Influence Pancreatic Cancer Outcomes. Cell.

[24] Chakladar J., Kuo S.Z., Castaneda G., Li W.T., Gnanasekar A., Yu M.A., Chang E.Y., Wang X.Q., Ongkeko W.M. (2020). The Pancreatic Microbiome Is Associated with Carcinogenesis and Worse Prognosis in Males and Smokers. Cancers.

[25] Zhang X.M., Zhang Z.Y., Zhang C.H., Wu J., Wang Y.X., Zhang G.X. (2018). Intestinal Microbial Community Differs between Acute Pancreatitis Patients and Healthy Volunteers. Biomed. Environ. Sci..

[26] Zhu Y., He C., Li X., Cai Y., Hu J., Liao Y., Zhao J., Xia L., He W., Liu L. (2019). Gut Microbiota Dysbiosis Worsens the Severity of Acute Pancreatitis in Patients and Mice. J. Gastroenterol..

[27] Frost F., Weiss F.U., Sendler M., Kacprowski T., Rühlemann M., Bang C., Franke A., Völker U., Völzke H., Lamprecht G. (2020). The Gut Microbiome in Patients with Chronic Pancreatitis Is Characterized by Significant Dysbiosis and Overgrowth by Opportunistic Pathogens. Clin. Transl. Gastroenterol..

[28] Adolph T.E., Mayr L., Grabherr F., Schwärzler J., Tilg H. (2019). Pancreas–Microbiota Cross Talk in Health and Disease. Annu. Rev. Nutr..

[29] Ciocan D., Rebours V., Voican C.S., Wrzosek L., Puchois V., Cassard A.-M., Perlemuter G. (2018). Characterization of Intestinal Microbiota in Alcoholic Patients with and without Alcoholic Hepatitis or Chronic Alcoholic Pancreatitis. Sci. Rep..

[30] Jandhyala S.M., Madhulika A., Deepika G., Rao G.V., Reddy D.N., Subramanyam C., Sasikala M., Talukdar R. (2017). Altered Intestinal Microbiota in Patients with Chronic Pancreatitis: Implications in Diabetes and Metabolic Abnormalities. Sci. Rep..

[31] Zhou W., Zhang D., Li Z., Jiang H., Li J., Ren R., Gao X., Li J., Wang X., Wang W. (2021). The Fecal Microbiota of Patients with Pancreatic Ductal Adenocarcinoma and Autoimmune Pancreatitis Characterized by Metagenomic Sequencing. J. Transl. Med..

[32] Yu S., Xiong Y., Xu J., Liang X., Fu Y., Liu D., Yu X., Wu D. (2020). Identification of Dysfunctional Gut Microbiota Through Rectal Swab in Patients with Different Severity of Acute Pancreatitis. Dig. Dis. Sci..

[33] Zhou C.H., Meng Y.T., Xu J.J., Fang X., Zhao J.L., Zhou W., Zhao J., Han J.C., Zhang L., Wang K.X. (2020). Altered Diversity and Composition of Gut Microbiota in Chinese Patients with Chronic Pancreatitis. Pancreatology.

[34] Half E., Keren N., Reshef L., Dorfman T., Lachter I., Kluger Y., Reshef N., Knobler H., Maor Y., Stein A. (2019). Fecal Microbiome Signatures of Pancreatic Cancer Patients. Sci. Rep..

[35] Kitsios G.D., Morowitz M.J., Dickson R.P., Huffnagle G.B., McVerry B.J., Morris A. (2017). Dysbiosis in the Intensive Care Unit: Microbiome Science Coming to the Bedside. J. Crit. Care.

[36] Besselink M.G., van Santvoort H.C., Buskens E., Boermeester M.A., van Goor H., Timmerman H.M., Nieuwenhuijs V.B., Bollen T.L., van Ramshorst B., Witteman B.J. (2008). Probiotic Prophylaxis in Predicted Severe Acute Pancreatitis: A Randomised, Double-Blind, Placebo-Controlled Trial. Lancet.

[37] Gou S., Yang Z., Liu T., Wu H., Wang C. (2014). Use of Probiotics in the Treatment of Severe Acute Pancreatitis: A Systematic Review and Meta-Analysis of Randomized Controlled Trials. Crit. Care.

[38] Hu Y., Xiao H.Y., He C., Lv N.H., Zhu L. (2019). Fecal Microbiota Transplantation as an Effective Initial Therapy for Pancreatitis Complicated with Severe *Clostridium difficile* Infection: A Case Report. World J. Clin. Cases.

[39] Li J.J., Zhu M., Kashyap P.C., Chia N., Tran N.H., McWilliams R.R., Bekaii-Saab T.S., Ma W.W. (2021). The Role of Microbiome in Pancreatic Cancer. Cancer Metastasis Rev..

[40] Yang J., Wang H., Lin X., Liu J., Feng Y., Bai Y., Liang H., Hu T., Wu Z., Lai J. (2024). Gut Microbiota Dysbiosis Induced by Alcohol Exposure in Pubertal and Adult Mice. mSystems.

[41] Clemens D.L., Wells M.A., Schneider K.J., Singh S. (2014). Molecular Mechanisms of Alcohol Associated Pancreatitis. World J. Gastrointest. Pathophysiol..

[42] Dell’Olio A., Scott W.T., Taroncher-Ferrer S., San Onofre N., Soriano J.M., Rubert J. (2024). Tailored Impact of Dietary Fibers on Gut Microbiota: A Multi-Omics Comparison on the Lean and Obese Microbial Communities. Microbiome.

[43] Portincasa P., Bonfrate L., Vacca M., De Angelis M., Farella I., Lanza E., Khalil M., Wang D.Q.-H., Sperandio M., Di Ciaula A. (2022). Gut Microbiota and Short Chain Fatty Acids: Implications in Glucose Homeostasis. Int. J. Mol. Sci..

[44] Kimble R., Gouinguenet P., Ashor A., Stewart C., Deighton K., Matu J., Griffiths A., Malcomson F.C., Joel A., Houghton D. (2023). Effects of a Mediterranean Diet on the Gut Microbiota and Microbial Metabolites: A Systematic Review of Randomized Controlled Trials and Observational Studies. Crit. Rev. Food Sci. Nutr..

[45] Mo C., Lou X., Xue J., Shi Z., Zhao Y., Wang F., Chen G. (2024). The Influence of Akkermansia muciniphila on Intestinal Barrier Function. Gut Pathog..

[46] Mann E.R., Lam Y.K., Uhlig H.H. (2024). Short-Chain Fatty Acids: Linking Diet, the Microbiome and Immunity. Nat. Rev. Immunol..

[47] Gebrayel P., Nicco C., Al Khodor S., Bilinski J., Caselli E., Comelli E.M., Egert M., Giaroni C., Karpinski T.M., Loniewski I. (2022). Microbiota Medicine: Towards Clinical Revolution. J. Transl. Med..

[48] Gibson G.R., Hutkins R., Sanders M.E., Prescott S.L., Reimer R.A., Salminen S.J., Scott K., Stanton C., Swanson K.S., Cani P.D. (2017). Expert Consensus Document: The International Scientific Association for Probiotics and Prebiotics (ISAPP) Consensus Statement on the Definition and Scope of Prebiotics. Nat. Rev. Gastroenterol. Hepatol..

[49] Singh A., Aggarwal M., Garg R., Stevens T., Chahal P. (2022). Post-Pancreatitis Diabetes Mellitus: Insight on Optimal Management with Nutrition and Lifestyle Approaches. Ann. Med..

[50] Phillips M.E., Hopper A.D., Leeds J.S., Roberts K.J., McGeeney L., Duggan S.N., Kumar R. (2021). Consensus for the Management of Pancreatic Exocrine Insufficiency: UK Practical Guidelines. BMJ Open Gastroenterol..

[51] Rangraze I.R., El-Tanani M., Wali A.F., Babiker R., Rabbani S.A., Matalka I.I., Satyam S.M., Avagimyan A., Hoffmann K., Ilias I. (2025). Type 5 Diabetes Mellitus: Redefining Pancreatogenic Diabetes through Molecular, Imaging, and AI-Driven Evidence. Front. Endocrinol..

[52] Arvanitakis M., Ockenga J., Bezmarevic M., Gianotti L., Krznarić Ž., Lobo D.N., Löser C., Madl C., Meier R., Phillips M. (2020). ESPEN Guideline on Clinical Nutrition in Acute and Chronic Pancreatitis. Clin. Nutr..

[53] American Diabetes Association Professional Practice Committee (2025). 5. Facilitating Positive Health Behaviors and Well-Being to Improve Health Outcomes: Standards of Care in Diabetes-2025. Diabetes Care.

[54] Vujasinovic M., Nikolic S., Gordon Achour A., Löhr J.M. (2023). Autoimmune Pancreatitis and Micronutrients. Dig. Liver Dis..

[55] Kaur H., Sharma A., Dimowo P.K., Rajeev A., Shaikh S.S., Mohammed L. (2026). Gut or Vein? Enteral Nutrition Versus Total Parenteral Nutrition in Acute Pancreatitis: A Systematic Review. Cureus.

[56] Qi D., Yu B., Huang J. (2018). Meta-Analysis of Early Enteral Nutrition Provided Within 24 Hours of Admission on Clinical Outcomes in Acute Pancreatitis. JPEN J. Parenter. Enter. Nutr..

[57] Bakker O.J., van Brunschot S., van Santvoort H.C., Besselink M.G., Bollen T.L., Boermeester M.A., Dejong C.H.C., van Goor H., Bosscha K., Ahmed Ali U. (2014). Early versus On-Demand Nasoenteric Tube Feeding in Acute Pancreatitis. N. Engl. J. Med..

[58] Wang X., Li W., Li N., Li J. (2008). Omega-3 Fatty Acids-Supplemented Parenteral Nutrition Decreases Hyperinflammatory Response and Attenuates Systemic Disease Sequelae in Severe Acute Pancreatitis: A Randomized and Controlled Study. JPEN J. Parenter. Enter. Nutr..

[59] Ul Ain Q., Bashir Y., Kelleher L., Bourne D.M., Egan S.M., McMahon J., O’Connor A., Ryan B.M. (2021). Dietary Intake in Patients with Chronic Pancreatitis: A Systematic Review and Meta-Analysis. World J. Gastroenterol..

[60] Wiese M., Gärtner S., Doller J., Tran Q.T., Frost F., Bannert K., Simon P., Klose S., Timm S., Mössner J. (2021). Nutritional Management of Chronic Pancreatitis: A Systematic Review and Meta-Analysis of Randomized Controlled Trials. J. Gastroenterol. Hepatol..

[61] Emanuel A., Krampitz J., Rosenberger F., Kind S., Rötzer I. (2022). Nutritional Interventions in Pancreatic Cancer: A Systematic Review. Cancers.

[62] Gianotti L., Besselink M.G., Sandini M., Hackert T., Conlon K., Gerritsen A., Griffin O., Hider S., Radenkovic D., Søreide K. (2018). Nutritional Support and Therapy in Pancreatic Surgery: A Position Paper of the International Study Group on Pancreatic Surgery (ISGPS). Surgery.

[63] Zhang Y., Zhang T., Yang W., Chen H., Geng X., Li G., Wang X., Liu Z., Zhang Y., Wang X. (2021). Beneficial Diets and Pancreatic Cancer: Molecular Mechanisms and Clinical Practice. Front. Oncol..

